# Effects and Mechanisms of a Web- and Mobile-Based Acceptance and Commitment Therapy Intervention for Anxiety and Depression Symptoms in Nurses: Fully Decentralized Randomized Controlled Trial

**DOI:** 10.2196/51549

**Published:** 2023-11-27

**Authors:** Yan'e Lu, Yang Li, Yongqi Huang, Xuan Zhang, Juan Wang, Liuliu Wu, Fenglin Cao

**Affiliations:** 1 Department of Nursing Psychology School of Nursing and Rehabilitation Shandong University Jinan China; 2 School of Nursing The University of Texas at Austin Austin, TX United States

**Keywords:** acceptance and commitment therapy, anxiety, depression, internet-based intervention, nurse, randomized controlled trial

## Abstract

**Background:**

Acceptance and commitment therapy (ACT) is a promising intervention for improving mental health. However, there is limited evidence on its effectiveness for nurses, particularly in web- and mobile-based intervention forms, in mitigating anxiety and depression symptoms.

**Objective:**

In this study, we aimed to examine the effect and underlying psychological mechanisms of a web- and mobile-based ACT intervention on nurses’ anxiety and depression symptoms.

**Methods:**

In this fully decentralized randomized controlled trial, nurses were recruited nationwide across China through advertisements and posters. They were randomly assigned to either the 5-week fully automated intervention or the waiting group. Primary outcomes (anxiety and depression symptoms); secondary outcomes (sleep quality, burnout, and work performance); and mediators (psychological flexibility, cognitive defusion, mindfulness, and values) were assessed using the Wenjuanxing platform. Data collectors were blinded to the group assignments throughout the study period.

**Results:**

A total of 145 nurses with anxiety or depression symptoms were randomly assigned to either the intervention group (n=72, 49.7%) or the control group (n=73, 50.3%); 97.2% (n=141) were female. During the study, 36 (24.8%) nurses were lost to follow-up, and 53 (73.6%) completed the entire intervention. Nurses in the intervention group showed significant improvement in anxiety (*d*=0.67, 95% CI 0.33-1.00) and depression symptoms (*d*=0.58, 95% CI 0.25-0.91), and the effects were sustained for 3 months after the intervention (anxiety: *d*=0.55, 95% CI 0.22-0.89; depression: *d*=0.66, 95% CI 0.33-1.00). Changes in psychological flexibility, cognitive defusion, and values mediated the effect of the intervention on anxiety and depression symptoms, while mindfulness did not have a mediating effect.

**Conclusions:**

The web- and mobile-based ACT intervention used in this study significantly improved nurses’ anxiety and depression symptoms by improving psychological flexibility, cognitive defusion, and values. The results provide new ideas for hospital administrators to prevent and intervene in nurses’ psychological issues.

**Trial Registration:**

Chinese Clinical Trial Register ChiCTR2200059218; https://tinyurl.com/4mb4t5y9

## Introduction

### Background

Nurses are at a high risk for anxiety and depression symptoms [1]. Especially during the COVID-19 pandemic, nurses were faced with a variety of challenges, including a dramatic increase in workload and shift frequency, physical strain, high risk of infection, and a shortage of health care workers, all of which contributed to more severe anxiety and depression symptoms [2]. A meta-analysis showed that the prevalence of anxiety and depression symptoms among nurses during the COVID-19 pandemic was 31.93% and 32.59%, respectively [2], which is higher than the prevalence among the general population [3]. These symptoms have been found to be associated with poor job satisfaction, nursing quality, work performance, and increased absenteeism and turnover among nurses [4-6]. Therefore, effective mental health interventions for nurses with anxiety and depression symptoms are urgently required. With the rapid development of the health care sector, innovative therapeutic approaches and digital platforms offer promising solutions [7]. Acceptance and commitment therapy (ACT) is one such approach that is gaining recognition [8].

ACT was initially developed in the late 1970s [9] and is considered the most prominent psychobehavioral therapy among the third generation of cognitive behavioral therapy [10]. Unlike traditional cognitive behavioral therapy, ACT emphasizes core values and does not seek to alter an individual’s abnormal thinking; instead, it focuses on changing the relationship between the individual and the symptom [11]. In recent years, ACT has been widely used to treat various mental disorders and psychological problems, particularly anxiety and depression symptoms, with satisfactory results [12,13], suggesting that it may be a feasible treatment for alleviating anxiety and depression symptoms in nurses.

Only a few studies have preliminarily examined the effects of ACT on nurses’ health outcomes. For instance, Frögéli et al [14] found that ACT intervention delivered in group meetings could prevent stress-related health problems among nursing students. A study with 35 health care workers showed that 1-day ACT group workshops effectively reduced their psychological distress [15]. Another study found that nurses in the ACT intervention group reported a significant reduction in the number of leaves taken due to injury and fewer mental health symptoms after the intervention than did the control group [16]. However, most of these studies had small sample sizes and involved face-to-face interventions. In China, nurses often face frequent overtime work, day and night shifts, a strict work scheduling system, and a heavy workload, which limit their free time [17]. Therefore, conducting face-to-face group interventions with nurses is extremely challenging.

Psychological interventions delivered via web- and mobile-based apps have many advantages over face-to-face group interventions [18]. First, web- and mobile-based interventions are convenient to implement and not limited by time, venue, or transportation. Second, these interventions offer greater privacy [7]; research has shown that nurses with psychological problems can be reluctant to seek medical treatment due to professional reasons or stigma [19] and that digital interventions that offer privacy reduce such concerns [7]. Third, web- and mobile-based interventions can be less costly and may not rely heavily on psychotherapists [20,21]. Fourth, web- and mobile-based interventions are highly accessible, as the number of internet users in China had reached 989 million in December 2020 [22]. Therefore, web- and mobile-based interventions may be better suited to the occupational characteristics of nurses. However, no studies have used a randomized controlled trial (RCT) design with a large sample to explore the effect of web- and mobile-based ACT interventions on anxiety and depression symptoms in nurses.

Furthermore, understanding the mechanisms by which psychological interventions work can help optimize patient treatment [23]. Mediation analysis is an important approach for investigating intervention mechanisms [24], as it can identify intervention components that may statistically explain potential associations between treatment and outcome variables [25]. According to the ACT psychotherapy model, ACT interventions aim to increase an individual’s psychological flexibility, an inherent ability to live in the moment, to be open to all experiences, and to act on what matters [10]. Vasiliou et al [26] found that ACT can improve the quality of life of patients with headache by increasing psychological flexibility. To increase psychological flexibility, ACT interventions target 6 interrelated psychological change processes, collectively known as the *hexaflex*, which includes contacting the present moment, cognitive defusion, mindful acceptance, self-as-context, clarifying values, and committed action [27]. Three of these processes, namely, cognitive defusion, mindfulness, and values, have received the most attention [28].

Cognitive defusion refers to creating a distance between one’s thoughts and oneself and is considered an effective way to reduce the impact of thoughts on behaviors [29]. Forman et al [30] found that ACT intervention works by reducing individuals’ cognitive fusion. Mindfulness, defined as intentional, nonjudgmental attention and awareness of the present moment, is viewed as an effective measure of helping individuals connect with the present moment and accept it [31]. One study found that mindfulness was the main mediator of the effect of ACT on depression symptoms [32]. In ACT, a value is likened to a compass that gives direction and guides individuals to move forward [33]. Clarifying values means a clear understanding of what is truly important to them, which is crucial for individuals to create a meaningful life and alleviate their psychological symptoms [24].

### Objectives

In summary, this study aimed to investigate the effects and mechanisms of a web- and mobile-based ACT intervention on anxiety and depression symptoms in nurses by using an RCT with a relatively large sample size. We hypothesized that the web- and mobile-based ACT intervention would lead to significant improvements in anxiety and depression symptoms among nurses, and psychological flexibility, cognitive defusion, mindfulness, and values might be the potential mechanisms for symptom reduction.

## Methods

### Study Design and Participants

This study was a fully decentralized, 2-arm, randomized, controlled trial with 1:1 allocation. The participants were divided into a web- and mobile-based ACT intervention group and a waiting control group. The research protocol was registered with the Chinese Clinical Trial Registry (ChiCTR2200059218). Nurses were recruited across China between April and August 2022 through advertisements and posters.

The inclusion criteria were as follows: the participants must (1) be aged ≥18 years, (2) have a college degree or higher, (3) hold a nurse practice certificate issued by the state, (4) have a Generalized Anxiety Disorder 7-item scale score of ≥5 or Patient Health Questionnaire-9 (PHQ-9) score of ≥5 (to identify nurses with mild or greater anxiety or depression symptoms for the intervention purpose), (5) not be undergoing any other psychological treatment or intervention in the past 6 months, (6) be able to use smartphones and WeChat, and (7) possess good reading and comprehension skills.

The exclusion criteria were (1) nurses who were not on duty due to sick leave, maternity leave, or sabbatical leave during the recruitment period; (2) those who were undergoing advanced training or practice; (3) nurses who were at risk of suicide, defined as a score of ≥2 on the suicidal ideation item of the PHQ-9; and (4) nurses who had previously completed a mindfulness or ACT course.

### Sample Size

The sample size was calculated using the G*Power (Heinrich-Heine-Universität Düsseldorf) software. A previous meta-analysis showed that the average improvement effect of ACT intervention on clinically relevant health problems could be moderate, with an effect size of 0.57 [13]. To achieve 85% test power with a 2-sided α of .05, we determined the estimated sample size to be 114. In addition, a systematic review of 20 studies that examined internet-delivered ACT for anxiety symptoms showed an attrition rate of 19.2% [34]. A recent meta-analysis of 64 studies found an overall dropout rate of 15.8% for ACT [35]. Thus, our study assumed an attrition rate of 17% (between 15% and 20%) and calculated a total sample size of 137, with a minimum of 69 participants required for each group.

### Procedures

Potential participants were recruited through advertisements. Interested nurses could scan a code to complete an electronic screening questionnaire. Those who met the study criteria were given detailed information about the format, content, process, and potential benefits and risks of the intervention. After consenting to participate, the nurses were randomized into either the intervention group or the waiting control group.

The web- and mobile-based ACT intervention was provided for 5 weeks to the intervention group, while the interventions were administered to the waiting control group after the completion of the study. Six questionnaires were administered at the following time points: baseline (T1: before the intervention); weekly (T2: week 2; T3: week 3; and T4: week 4); after the intervention (T5: week 5); and follow-up (T6: 3 months after the intervention). All assessments were conducted on Wenjuanxing, an online survey platform. The primary outcome variables (anxiety and depression symptoms) were evaluated at all 6 time points, whereas the secondary outcomes were assessed at T1, T5, and T6, and the intervention mechanism variables (psychological flexibility, cognitive defusion, mindfulness, and values) were assessed at T1 and T5.

The participants were randomized using a simple randomization method. Researchers who were not involved in the study numbered them, generated random sequences on a website, and then assigned participants to the appropriate group. The participants knew when they were participating in the intervention but were not aware of the group they were in. In addition, data collectors were unaware of the group assignments throughout the study period.

### Web- and Mobile-Based ACT Intervention

The intervention implemented in this study was adapted from the 2 ACT handbooks by Harris [33] and Hayes et al [36] by the members of the research team with ACT expertise. The intervention was adjusted based on the following principles: (1) targeting anxiety and depression symptoms in nurses, (2) tuning all metaphors and practices in the scheme to align with the characteristics of the nursing profession, and (3) making the interventions easy to understand and brief to learn.

The intervention lasted for 5 weeks and consisted of 5 modules, each covering a different theme: opening ACT, observing one’s mind, mindful living, knowing what matters, and doing important things. Each module was made available every Monday and included 2 parts: the thematic course and homework. The thematic course was divided into an overview of the content and a role-play of the practice, presented in the form of a video that was 15 to 30 minutes long. Participants were required to complete the thematic course within a week. Homework assignments were divided into worksheets and experiential exercises. The former consisted of inspirational and guiding questions regarding the content of the thematic course, while the latter comprised several mindfulness practices with audio recordings that were each 10 to 20 minutes long and could be practiced by participants multiple times (Multimedia Appendix 1). No support or guidance was provided to the participants during the intervention, other than responses to anonymous questions for each module.

The intervention was implemented through the Rain Classroom app, which is a WeChat app that provides online teaching and learning services (Multimedia Appendix 2) [37]. Participants could engage in the intervention on any device, such as desktop or laptop computers, smartphones, or tablets, as long as the WeChat app was installed. Rain Classroom is popular in China, particularly in universities and hospitals, owing to its user-friendly interface, various teaching forms, rich functions, and panoramic teaching data monitoring. Its advantages include free and easy access, real-time monitoring of participants’ learning progress, and the ability to release course learning reminders at any time. As of April 2021, Rain Classroom had provided online teaching to 43 million teachers and students in China [38], demonstrating its convenience and accessibility as an intervention tool. Every Monday during the intervention period, standardized information reminders were given to all participants in the intervention group; and every Thursday and weekend, personalized reminders were given to participants who did not complete the thematic course to improve intervention adherence. Participants were considered to have completed the intervention if they had completed the thematic courses of all modules in their entirety.

### Measures

#### Overview

General information consisted of the nurses’ demographic and work-related data. Demographic data included age, sex, BMI, average monthly income, marital status, and education level. Work-related data included work department, employment type, type of nursing role, position, working years, weekly working hours, and number of night shifts per month.

#### Primary Outcomes

The Generalized Anxiety Disorder 7-item scale was used to assess anxiety symptoms over the past 2 weeks in nurses [39]. The scale consists of 7 items, each rated on a 4-point Likert scale ranging from 0 (never) to 3 (nearly every day). The total score ranges from 0 to 21, with higher scores representing more severe anxiety. Following previous research [40], we adopted a cutoff score of 5 to identify nurses with mild or greater anxiety symptoms for the intervention purpose. In this study, the Cronbach α coefficient at baseline was .853.

The PHQ-9 was used to assess nurses’ depression symptoms over the past 2 weeks [41]. The scale has 9 items, and each item is scored on a Likert 4-point scale ranging from 0 (never) to 3 (nearly every day). The total score ranges from 0 to 27, with higher scores indicating more severe depression. Consistent with previous literature [42], this study used a cutoff score of 5 to indicate positive depression symptoms and intervene with nurses exhibiting mild or greater depression symptoms. In this study, the Cronbach α coefficient at baseline was .767.

#### Secondary Outcomes

The Pittsburgh Sleep Quality Index scale [43] was used to assess the sleep quality of nurses over the past month. There are 19 items in the scale, with each item being rated on a 4-point scale ranging from 0 (no difficulty) to 3 (severe difficulty). The total score ranges from 0 to 21, with higher scores indicating poorer sleep quality. In this study, the Cronbach α coefficient at baseline was .715.

The Maslach Burnout Inventory-Human Services Survey for Medical Personnel [44] scale was specifically designed for use in the service and health care industry and was used to assess burnout in nurses. The scale comprises 22 items, and each item is rated on a 7-point Likert scale ranging from 0 (never) to 6 (every day). Higher scores on the total scale represent higher levels of burnout among nurses. In this study, the Cronbach α coefficient at baseline was .779.

The Work Performance Scale, translated and revised by a Taiwanese scholar, was used to assess nurses’ work performance [45]. The scale consists of 11 items, each rated on a 5-point Likert scale ranging from 1 (strongly disagree) to 5 (strongly agree). Higher total scores indicate higher work performance levels among nurses. In this study, the Cronbach α coefficient at baseline was .852.

#### Mediators

The Acceptance and Action Questionnaire, second edition, was used to assess the psychological flexibility of nurses [46]. The scale consists of 7 items, with each item scored on a 7-point Likert scale ranging from 1 (never) to 7 (always). The total score ranges from 0 to 49. The higher the total score, the higher the level of individual experiential avoidance, which indicates lower levels of psychological flexibility. In this study, the Cronbach α coefficient at baseline was .916.

The Cognitive Fusion Questionnaire [47] was used to assess cognitive defusion among the nurses. There are 9 items on the scale, and each item is scored on a 7-point Likert scale ranging from 1 (never) to 7 (always). The total score ranges from 0 to 63, which is the sum of all items. Higher total scores indicate higher levels of cognitive fusion and lower levels of cognitive defusion. In this study, the Cronbach α coefficient at baseline was .966.

The Mindful Attention Awareness Scale [48] was used to assess nurses’ mindfulness levels. The scale has 15 items, and each item is rated on a Likert 6-point scale from 1 (almost always) to 6 (almost never). The total score is the sum of all items, and a higher total score represents a higher level of awareness and attention to the present state in daily life, that is, a higher level of mindfulness. In this study, the Cronbach α coefficient at baseline was .911.

The values of nurses were assessed using the Valuing Questionnaire [49]. The scale has 10 items and is divided into 2 dimensions: progress and obstacle. The progress dimension reflects the construction of individual values, such as whether an individual can realize what is most important to them and the extent to which they adhere to the direction of their values in action**.** The obstacle dimension reflects the destruction of valuable life, such as the avoidance of distressing experiences and neglect of values in action. Each item is rated on a 7-point Likert scale from 0 (not at all) to 6 (fully), and the sum of all items for each dimension is the score for each dimension. Higher scores for each dimension represent higher levels of individuals in that dimension. In this study, the Cronbach α coefficient at baseline was .837 for the progress dimension and .743 for the obstacle dimension.

### Ethical Considerations

The study design and procedures were in accordance with the Declaration of Helsinki. This study was approved by the Ethics Review Committee of the School of Nursing and Rehabilitation of Shandong University (2022-R-60). Participation was voluntary, and participants could withdraw from the study at any time. All participants provided informed consent before participation.

### Statistical Analysis

Data management and statistical analysis were performed using SPSS (version 26.0; IBM Corp), and a 2-sided α of <.05 was used to determine statistical significance. Normality tests were conducted for all continuous variables, and the results showed that all continuous variables in this study were normally or approximately normally distributed. Therefore, mean and SD was used to describe continuous variables. Frequency (percentage) was used to describe the categorical variables. Chi-square tests were used to compare baseline general information between participants who completed the questionnaire and those who did not complete throughout the intervention and follow-up.

The generalized estimating equation (GEE) was used to assess the group, time, and time×group effects of the intervention on primary (anxiety and depression symptoms) and secondary outcomes (sleep quality, burnout, and work performance) in nurses. GEE has been widely used in the analysis of repeated measured randomized controlled design data and allows for missing values in the data, differences in the number of observations for each observation object, and the time interval between observations [50,51]. The primary analysis was based on the intention-to-treat analysis principle. To assess the robustness of the primary analysis results, sensitivity analyses were performed using cases with complete data at all time points (Multimedia Appendix 3). Effect sizes were calculated by dividing the difference between the 2 groups by the combined SD. Small, medium, and large effect sizes were considered as *d*=0.2, 0.5, and 0.8, respectively, and the 95% CI of the effect size excluding 0 was considered as statistically significant.

Model 4 in the PROCESS plug-in was used to test the mediating effect of the intervention on nurses’ anxiety and depression symptoms. First, the residualized change scores of the mechanism variables and primary outcome variables were calculated based on the linear regression model to represent the changes in individuals before (T1) and after (T5) the intervention. The residualized change score was used as the mediating variable and outcome variable for the mediating analysis. The residualized change score represents the difference between an individual’s actual score immediately after the intervention and the score predicted at baseline [52]. Unlike the original change score measured before and after the intervention (T5-T1), residual change scores accounted for the confounding effects of individual baseline scores, controlling for correlations between pre- and postintervention scores. This facilitates attempts to account for temporality in the mediation model, an important aspect when examining causality [53,54]. The nonparametric percentile bootstrap method for deviation correction (n=5000) was used to test for mediation effects, and a significant mediation effect was implied if the 95% CI for the indirect effect did not contain 0.

## Results

### Participant Recruitment and Dropout

**Figure 1:**
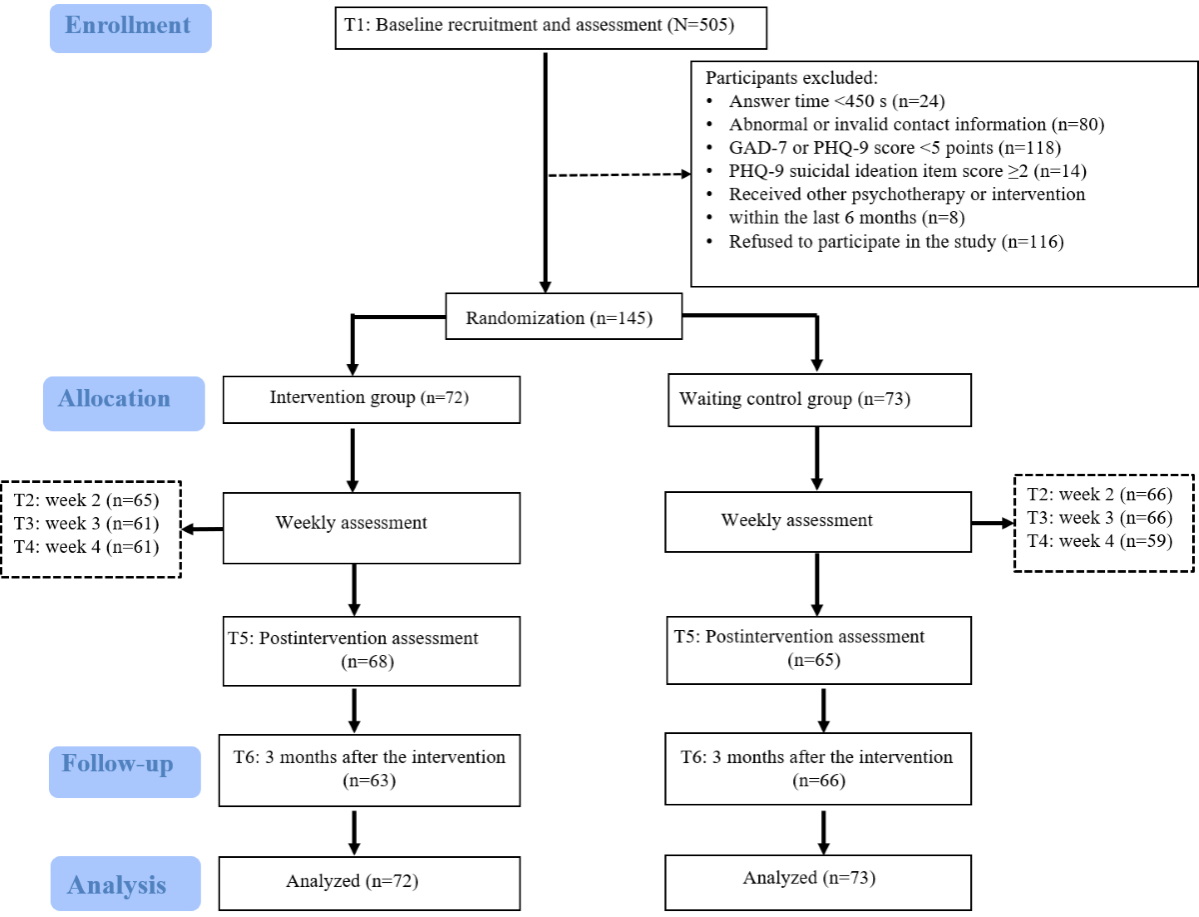
Flow diagram of intervention for anxiety and depression symptoms in nurses. GAD-7: Generalized Anxiety Disorder 7-item scale; PHQ-9: Patient Health Questionnaire-9.

A total of 505 nurses were recruited, and 145 nurses were included and randomly assigned to either the intervention group or the control group (Figure 1). Of them, 72 (49.7%) were placed in the intervention group and 73 (50.3%) were placed in the control group. Participants who did not complete the questionnaire assessment at any of the T1 to T6 measures were considered lost to follow-up. During the study period, 36 (24.8%) nurses were lost to follow-up. However, there were no statistical differences in baseline general information between the completed sample and the missing sample (Multimedia Appendix 4).

### Participant Baseline Characteristics and Adherence

The mean age of participants at baseline was 35.36 (SD 7.22) years, and 97.2% (141/145) were female participants. Their mean scores for anxiety and depression symptoms were 8.40 (SD 3.75) and 10.57 (SD 4.27), respectively (Table 1). In the intervention group (n=72), 62 (86%) participants completed at least 3 of the 6 sessions (ie, half of the course), and 53 completed the entire course, resulting in an intervention completion rate of 74% (53/72). The average time taken to complete the intervention was 4.28 (SD 1.43) weeks.

**Table 1:** Baseline characteristics of participants.

Variable			Total sample (N=145)		Control group (n=73)		Intervention group (n=72)
**Age (y), n (%)**							
	<35	65 (44.8)		29 (39.7)		36 (50)	
	≥35	80 (55.2)		44 (60.3)		36 (50)	
**Sex, n (%)**							
	Male	4 (2.8)		2 (2.7)		2 (2.8)	
	Female	141 (97.2)		71 (97.3)		70 (97.2)	
**BMI (kg/m^2^), n (%)**							
	Normal (18.5-23.9)	94 (64.8)		52 (71.2)		42 (58.3)	
	Abnormal (<18.5 or ≥24)	51 (35.2)		21 (28.8)		30 (41.7)	
**Average monthly income** **(yuan; 1 yuan=US $0.1402), n (%)**							
	<8000	63 (43.4)		28 (38.4)		35 (48.6)	
	≥8000	82 (56.6)		45 (61.6)		37 (51.4)	
**Marital status, n (%)**							
	Single	31 (21.4)		16 (21.9)		15 (20.8)	
	Married	114 (78.6)		57 (78.1)		57 (79.2)	
**Education, n (%)**							
	Bachelor’s or lower degree	122 (84.1)		61 (83.6)		61 (84.7)	
	Master’s or higher degree	23 (15.9)		12 (16.4)		11 (15.3)	
**Work department, n (%)**							
	Internal medicine	48 (33.1)		21 (28.8)		27 (37.5)	
	Surgery	24 (16.6)		9 (12.3)		15 (20.8)	
	Gynecology and pediatrics	14 (9.7)		7 (9.6)		7 (9.7)	
	Other	59 (40.7)		36 (49.3)		23 (31.9)	
**Employment type, n (%)**							
	Authorized strength	71 (49)		36 (49.3)		35 (48.6)	
	Human agency	15 (10.3)		11 (15.1)		4 (5.6)	
	Other	59 (40.7)		26 (35.6)		33 (45.8)	
**Type of nursing role, n (%)**							
	Nurse practitioner or lower	58 (40)		27 (37)		31 (43.1)	
	Supervisor or higher	87 (60)		46 (63)		41 (56.9)	
**Position, n (%)**							
	Nurse	109 (75.2)		52 (71.2)		57 (79.2)	
	Head nurse or higher	36 (24.8)		21 (28.8)		15 (20.8)	
**Working years, n (%)**							
	≤10	66 (45.5)		30 (41.1)		36 (50)	
	＞10	79 (54.5)		43 (58.9)		36 (50)	
**Weekly working hours per week, n (%)**							
	≤40	36 (24.8)		16 (21.9)		20 (27.8)	
	>40	109 (75.2)		57 (78.1)		52 (72.2)	
**Number of night shifts per month, n (%)**							
	0	64 (44.1)		34 (46.6)		30 (41.7)	
	1-4	27 (18.6)		12 (16.4)		15 (20.8)	
	≥5	54 (37.2)		27 (37)		27 (37.5)	
Anxiety symptom score, mean (SD)			8.40 (3.75)		8.47 (3.81)		8.33 (3.72)
Depression symptom score, mean (SD)			10.57 (4.27)		10.58 (4.08)		10.56 (4.49)
Sleep quality score, mean (SD)			10.68 (2.66)		10.67 (2.76)		10.69 (2.57)
Job burnout score, mean (SD)			53.24 (21.06)		53.64 (21.47)		52.83 (20.77)
Work performance score, mean (SD)			43.70 (6.08)		43.62 (5.79)		43.78 (6.39)
Psychological flexibility score, mean (SD)			27.59 (7.94)		27.25 (8.05)		27.94 (7.87)
Cognitive defusion score, mean (SD)			37.89 (11.17)		37.27 (10.76)		38.50 (11.61)
Mindfulness score, mean (SD)			49.64 (11.84)		50.23 (11.58)		49.04 (12.16)
Values progress score, mean (SD)			17.58 (5.01)		17.63 (4.58)		17.53 (5.44)
Values obstacle score, mean (SD)			16.03 (4.84)		16.32 (4.73)		15.74 (4.97)

### Intervention Effect on Outcomes

#### Primary Outcomes

**Figure 2:**
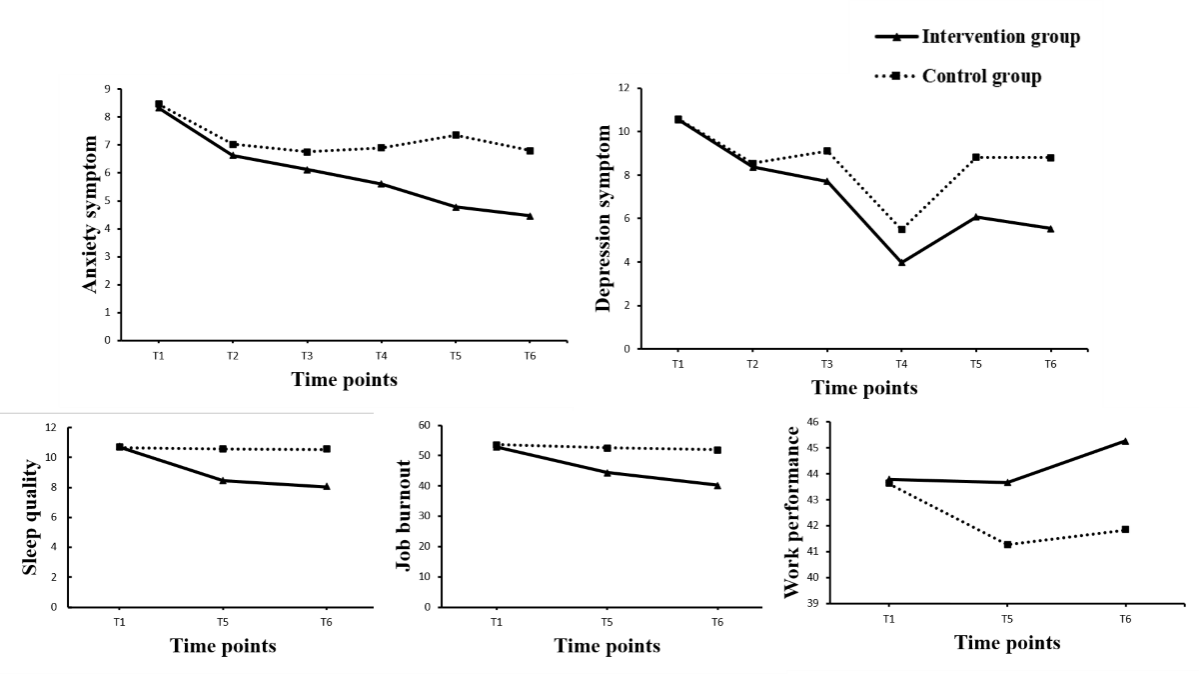
Generalized estimating equation–estimated outcome variable score changes over time for the intervention and control groups. T1: baseline; T2: week 2; T3: week 3; T4: week 4; T5: after the intervention; T6: 3 months after the intervention.

As shown in Table 2, the GEE model for anxiety symptoms revealed a statistically significant group effect, time effect, and group×time interaction effect, indicating the effectiveness of the intervention. The intergroup effect sizes at T5 and T6 time points were 0.67 (95% CI 0.33-1.00) and 0.55 (95% CI 0.22-0.89), respectively. Over time, the anxiety symptom scores of the intervention group continued to decline, while those of the control group declined slowly and remained almost stable after T2 (Figure 2).

The group effect, time effect, and group×time interaction effect were statistically significant in the GEE model for depression symptoms, indicating the effectiveness of the intervention (Table 2). The intergroup effect sizes at T5 and T6 were 0.58 (95% CI 0.25-0.91) and 0.66 (95% CI 0.33-1.00), respectively. The depression symptom scores in the intervention group decreased to a minimum at T4, rebounded at T5, and remained stable at T6. In the control group, depression symptom scores decreased at T2, increased slightly at T3, dropped to a minimum at T4, and then rebounded, remaining stable at T5 and T6 (Figure 2).

**Table 2:** Effect of intervention on primary and secondary outcomes in nurses.

Outcome and time			Estimated mean difference (95% CI)		*P* value		Cohen *d* (95% CI)		Group effect							Time effect							Group×time effect			
									Wald *χ*^*2*^			*P* value			Wald *χ*^*2*^				*P* value			Wald *χ*^*2*^			*P* value	
**Anxiety symptoms**										5.66			.02^a^				61.36			<.001^a^			20.50			.001^a^
	T1^b^	−0.13 (−1.35 to 1.08)		.83		Reference^c^																				
	T2^d^	−0.40 (−1.59 to 0.79)		.51		0.11 (−0.21 to 0.44)																				
	T3^e^	−0.64 (−1.95 to 0.66)		.34		0.17 (−0.15 to 0.50)																				
	T4^f^	−1.29 (−2.69 to 0.11)		.07		0.33 (0.00 to 0.66)																				
	T5^g^	−2.57 (−0.87 to −3.35)		<.001^a^		0.67 (0.33 to 1.00)																				
	T6^h^	−2.34 (−3.78 to −0.90)		.001^a^		0.55 (0.22 to 0.89)																				
**Depression symptoms**										6.95			.008^a^				401.03			<.001^a^			21.29			.001^a^
	T1	−0.02 (−1.41 to 1.37)		.98		Reference																				
	T2	−0.17 (−1.59 to 1.24)		.81		0.04 (−0.28 to 0.37)																				
	T3	−1.40 (−3.07 to 0.27)		.10		0.29 (−0.04 to 0.62)																				
	T4	−1.51 (−2.61 to −0.41)		.007^a^		0.49 (0.16 to 0.82)																				
	T5	−2.74 (−4.34 to −1.14)		.001^a^		0.58 (0.25 to 0.91)																				
	T6	−3.26 (−4.94 to −1.58)		<.001^a^		0.66 (0.33 to 1.00)																				
**Sleep quality**										14.24			<.001^a^				37.91			<.001^a^			30.76			<.001^a^
	T1	0.03 (−0.84 to 0.89)		.95		Reference																				
	T5	−2.11 (−3.06 to −1.15)		<.001^a^		0.75 (0.41 to 1.08)																				
	T6	−2.50 (−3.56 to −1.44)		<.001^a^		0.81 (0.47 to 1.15)																				
**Job burnout**										5.25			.02^a^				17.92			<.001^a^			10.53			.005^a^
	T1	−0.81 (−7.64 to 6.02)		.82		Reference																				
	T5	−8.23 (−15.35 to −1.11)		.02^a^		0.39 (0.06 to 0.71)																				
	T6	−11.80 (−18.61 to −4.98)		.001^a^		0.59 (0.26 to 0.92)																				
**Work performance**										4.08			.04^a^				4.92			.09			8.46			.02^a^
	T1	0.16 (−1.81 to 2.13)		.87		Reference																				
	T5	2.40 (−0.12 to 4.92)		.06		0.32 (0.00 to 0.65)																				
	T6	3.44 (0.67 to 6.20)		.02^a^		0.43 (0.10 to 0.76)																				

^a^The *P* value was statistically significant.

^b^T1: baseline.

^c^Effect size of the intervention was 0.

^d^T2: week 2.

^e^T3: week 3.

^f^T4: week 4.

^g^T5: after the intervention.

^h^T6: 3 months after the intervention.

#### Secondary Outcomes

The GEE model showed that sleep quality and job burnout scores in the intervention group were significantly lower than those in the control group at T5 and T6. In contrast, the work performance scores were significantly higher in the intervention group than in the control group at T6. Notably, the intergroup effect sizes for sleep quality at T5 and T6 were 0.75 (95% CI 0.41-1.08) and 0.81 (95% CI 0.47-1.15), respectively, indicating medium to large effect sizes (Figure 2 and Table 2).

### Mediators of Intervention Effect

The results of the mediation analysis found that the intervention group was negatively associated with both anxiety (B=−0.706; *P*<.001) and depression (B=−0.645; *P*<.001) symptom residuals among nurses. As shown in Table 3, changes in psychological flexibility, cognitive defusion, and value progress and obstacle all had a mediating effect on the improvement of nurses’ anxiety and depression symptoms through the intervention. However, no mediating effect of mindfulness on anxiety and depression symptoms was observed after the intervention.

**Table 3:** Effect of mediators of the intervention on anxiety and depression symptoms.

Variable			Direct effect				Indirect effect (95% CI)
			B (SE)		*P* value		
**Outcome: anxiety symptom residual**							
	Psychological flexibility residual	−0.439 (0.100)		.003		−0.267 (−0.447 to −0.984)	
	Cognitive defusion residual	−0.363 (0.144)		.01		−0.343 (−0.554 to −0.161)	
	Mindfulness residual	−0.569 (0.149)		<.001		−0.137 (−0.273 to 0.001)	
	Value progress residual	−0.564 (0.160)		<.001		−0.142 (−0.326 to −0.028)	
	Value obstacle residual	−0.565 (0.161)		<.001		−0.141 (−0.299 to −0.036)	
**Outcome: depression symptom residual**							
	Psychological flexibility residual	−0.414 (0.161)		.01		−0.231 (−0.431 to −0.074)	
	Cognitive defusion residual	−0.400 (0.170)		.02		−0.245 (−0.400 to −0.115)	
	Mindfulness residual	−0.532 (0.166)		.002		−0.113 (−0.258 to 0.000)	
	Value progress residual	−0.482 (0.172)		.006		−0.163 (−0.357 to −0.035)	
	Value obstacle residual	−0.519 (0.175)		.004		−0.127 (−0.287 to −0.022)	

## Discussion

### Principal Findings

To the best of our knowledge, this study is the first to examine the improvement effects and mechanisms of web- and mobile-based ACT interventions on anxiety and depression symptoms in nurses. The results showed that nurses in the intervention group reported significant improvement in anxiety and depression symptoms after the intervention compared with the waiting control group, and the improvement persisted for 3 months after the intervention. The web- and mobile-based ACT intervention also demonstrated the potential to improve nurses’ sleep quality, burnout, and work performance. In addition, the results of mediation analysis demonstrated that psychological flexibility, cognitive defusion, and values served as mediators of the effect of the intervention on the reduction of anxiety and depression symptoms in nurses. However, no mediating effect of mindfulness was observed.

There are few studies on ACT interventions for nurses with anxiety or depression symptoms. A study involving 22 intensive care unit medical and nursing staff found that ACT-based stress management training significantly improved participants’ levels of depression symptoms (effect size=0.280), but not anxiety symptoms (effect size=0.069) [55]. Another recent study conducted during the COVID-19 outbreak showed that a group-based ACT intervention improved mental health symptoms, including anxiety and depression, among clinical nurses [56]. However, the first study had a small sample size and might have had insufficient statistical power, while the second was a nonrandomized, controlled, quasi-experimental study that did not report the effect size of the intervention. Using an RCT with multiple points of measurement in a larger sample of 143 participants, this study found that web- and mobile-based ACT interventions had substantial intervention effects on reducing nurses’ anxiety and depression symptoms, achieving moderate to large effect sizes.

Dropout rates and adherence are essential indicators of how well the study design aligns with participants’ needs and expectations, especially in the context of mental health among health care professionals. The high adherence in this study indicates that web- and mobile-based ACT interventions are feasible and acceptable for nurses. However, the dropout rate in this study was higher than the average dropout rate of 19.19% reported in a systematic review of internet-delivered ACT treatment for anxiety symptoms [34]. It is worth noting that participants had to complete the questionnaire assessment on Sundays, which conflicted with the schedule of the nurses on duty that day. Therefore, some participants completed the intervention but only participated in some of the 6 assessments, leading to the higher dropout rate. Nevertheless, ACT intervention delivered via the free Rain Classroom WeChat app may be promising for improving nurses’ health and well-being given its high convenience, high privacy, low cost, and feasibility features. Health care providers could potentially integrate a web- and mobile-based ACT intervention into nurses’ professional development and wellness programs, allowing them to access this free mental health self-help support on demand. This could be particularly beneficial in resource-limited countries with scarce mental health resources. In addition, policy makers should also consider the early identification and timely resolution of mental health problems among nurses.

In this study, we found that psychological flexibility mediated the improvement of anxiety and depression symptoms in nurses through the web- and mobile-based ACT intervention, and this finding is consistent with the theoretical framework of ACT and supports previous research results [32,57]. Psychological flexibility has been found to be a more effective strategy for emotion regulation than experiential avoidance, allowing nurses to reduce anxiety and depression symptoms by accepting painful thoughts, emotions, and feelings instead of overavoiding or controlling them in a way that leads to inflexibility [58].

Cognitive fusion occurs when individuals become so consumed by their thoughts that unhelpful thoughts dominate their behavior, leading to increased distress (sadness, anxiety, anger, depression, etc); limited range of behavior; and reduced ability to live a meaningful life [24]. Research has shown that cognitive fusion is positively associated with the development of negative emotional states [58]. In this study, we found that a decrease in cognitive fusion (increase in cognitive defusion) was a mediating factor for the effect of the intervention on anxiety and depression symptoms, and this finding aligns with that of the previous research [59]. One possible explanation for this finding is that web- and mobile-based ACT interventions can effectively help nurses acquire cognitive defusion skills, enabling them to step back and observe their own thoughts when faced with repeated negative thinking and thoughts that impede value-oriented living; by distinguishing between thoughts and facts in a timely manner, they could alleviate the adverse emotions generated by negative cognition and automatic thinking [60].

One of the main goals of ACT is to help individuals clarify their values and lead a rich, fulfilling, and meaningful life while accepting inevitable pain in life [24]. In this study, both value progress and obstacle dimensions were found to mediate the effects of the intervention on nurses’ anxiety and depression symptoms, and this is consistent with the findings of Lundgren et al [61]. Value progress reflects the construction of individual values, such as whether an individual can realize what matters and how much they adhere to their values in action, while the value obstacle represents the destruction of valuable life, such as avoidance of painful experiences and neglect of values when acting [49]. Studies have shown that value progress is associated with positive mental health outcomes such as well-being and life satisfaction, while value obstacles are associated with negative outcomes such as anxiety and depression symptoms [62]. In this study, the intervention could improve nurses’ value progress and reduce their value obstacles, thus promoting effective value-based actions. Studies based on self-determination theory and the ACT clinical model have shown that acting in line with one’s values is key to maintaining mental health, as opposed to impulsive, inactive, or ineffective actions [63,64].

Notably, no mediating effect of mindfulness was found in the intervention for improving nurses’ anxiety and depression symptoms, and this is inconsistent with previous research results [32,65]. One possible explanation for this inconsistency is that the thematic course in this study mainly focused on providing theoretical knowledge and demonstrating core skills such as cognitive defusion, clarifying values, and setting values-based goals, while mindfulness exercises were primarily incorporated into weekly homework. The adherence indicator for this study was defined as the completion of the entire 5-week thematic course, and participants were encouraged rather than required to complete homework; this might have resulted in inadequate mindfulness practice hours and insufficient improvement in mindfulness levels to test for mediating effects. Research has shown that the duration of mindfulness practice is positively correlated with its effectiveness [66].

### Limitations

This study has several potential limitations. First, all mediating and outcome variables were subjectively reported by the participants through questionnaires, which may have led to recall bias. Second, most participants reported a higher socioeconomic status, thereby limiting the generalizability of the study’s findings to disadvantaged nurses. In addition, our sample of nurses consisted of mostly female participants (97.24%). Although this study was not designed to analyze differences by sex, this skew is an important consideration for the generalizability of the results. Future studies should recruit more male nurses and perform subgroup analyses. Third, this participants were followed up only for 3 months after the intervention, which may limit the extent of the results. Thus, we recommend that future studies explore the impact of web- and mobile-based ACT interventions on nurses’ health outcomes over a more extended period to determine the extent to which improvements persist. Fourth, although there were no statistical differences in the baseline characteristics between the completed and missing samples, dropouts can introduce bias and affect the generalizability of the results. Similar future studies could explore potential strategies to reduce dropout rates. Fifth, all mediated analyses used only baseline versus immediate postintervention changes. Future studies could use more complex designs, such as intensive longitudinal mediated designs, to examine the effects of dynamic changes in ACT process variables on the relationship between intervention and outcomes at different time points [67].

### Conclusions

This study was the first to investigate the effects of web- and mobile-based ACT interventions on nurses’ anxiety and depression symptoms by using an RCT with a large sample size. Our findings are promising, demonstrating the effectiveness of the intervention on nurses’ health outcomes and providing new ideas for hospital administrators to prevent and intervene in nurses’ psychological issues. In addition, we comprehensively explored the psychological mechanism of web- and mobile-based ACT interventions and found that psychological flexibility, cognitive defusion, and values are the key mediating variables. This finding supports the *Hexaflex* treatment model of ACT, helps researchers narrow down the causal mechanisms of ACT web- and mobile-based intervention, and optimizes the treatment of anxiety and depression symptoms in nurses. Furthermore, our intervention is brief, engaging, and accessible through WeChat, without requiring the download of additional software. This feature is ideal for relieving stress in nurses who have heavy workloads and irregular free time due to shifts. Finally, the acceptable attrition rate and high adherence in this study further support the potential of the widely used WeChat app as an intervention delivery platform.

## Appendix

Multimedia Appendix 1 Web- and mobile-based acceptance and commitment therapy intervention program for anxiety and depression symptoms in nurses.

Multimedia Appendix 2 Screenshot of the Rain Classroom WeChat mini program.

Multimedia Appendix 3 Effects of the web- and mobile-based acceptance and commitment therapy intervention based on complete case data.

Multimedia Appendix 4 Comparison of baseline general information for completed and missed samples.

Multimedia Appendix 5 CONSORT-eHEALTH checklist (V 1.6.1).

## Abbreviations

ACT: acceptance and commitment therapy
GEE: generalized estimating equation
PHQ-9: Patient Health Questionnaire-9
RCT: randomized controlled trial

## Data Availability

The data sets generated during and/or analyzed during this study are not publicly available as they contain participant information, but are available from the corresponding author on reasonable request.
