# Supplementary material for: Effects and Mechanisms of a Web- and Mobile-Based Acceptance and Commitment Therapy Intervention for Anxiety and Depression Symptoms in Nurses: Fully Decentralized Randomized Controlled Trial
Source: J Med Internet Res. 2023 Nov 27;25:e51549. doi: 10.2196/51549 (PMC10714267; doi:10.2196/51549)
Supplement: Multimedia Appendix 1 [file jmir_v25i1e51549_app1.docx]

**Multimedia Appendix 1**

Web-based and mobile acceptance and commitment therapy intervention program for anxiety and depression symptoms in nurses.

| **Module/**  **week** | **Theme** | **Thematic course** | **Homework** | |
| --- | --- | --- | --- | --- |
|  |  | **Intervention content** | **Worksheet exercise** | **Experiential exercise** |
| 1 | **Opening ACT** | **Opening ACT**  **1.** **Overview：**  (1) Introduce the definition and goals of ACT.  (2) Introduce the six core pathologies and treatment processes of ACT, and visually explain the ACT model using the “demons on the boat” metaphor.  (3) Introduce the manifestations and dangers of anxiety and depression symptoms, and explain the role of ACT in managing anxiety and depression symptoms.  **2.** **Practice**：  Use the “folder” metaphor practice to guide nurses in active participation and reflection on the ACT experience.  **Video duration: 30 minutes.** | **Attempted solutions and their long-term effects：**  Nurses are encouraged to make a list of coping strategies they have taken in the last month to avoid or get rid of painful thoughts, feelings and sensations, and to answer the following questions.  (1) Have the thoughts or feelings that bothered me disappeared?  (2) Have these strategies made me more energetic or feel better in my work and life? | “Hexaflex” exercise;  Mindful Breathing. |
| 2 | **Observing your mind** | **Cognitive defusion**  **1.** **Overview：**  (1) Introduce the concepts of cognitive fusion and defusion, and illustrate them using the metaphor “the mind is like a radio”;  (2) Introduce common cognitive fusion situations and provide guidance on when to implement cognitive defusion techniques to combat it.  **2.** **Practice：**  Guide nurses through “taste defusion” and “repeatedly shouting out ideas” exercises to facilitate their ability to observe and distance themselves from unhelpful thoughts that may hinder them from living a values-based life.  **Self-as-context**  **1. Overview：**  (1) Introduce the concept of self-as-context and explain it using the metaphor “sky and weather”;  (2) Explain the difference between the observing self and the conceptualized self.  **2. Practice：**  Facilitate a “Continuous You” exercise for nurses, aimed at cultivating an awareness of the observing self and practicing cognitive defusion from the conceptualized self.  **Video duration: 30 minutes.** | **Idea defusion worksheet**：  Nurses are encouraged to list the trigger events or situations that cause cognitive integration and answer the following questions.  (1) What does my mind say or do to manipulate me?  (2) How does my behavior change when I am manipulated by thoughts?  (3) Do I try to free myself from the manipulation of my thoughts? If so, what did I do? | “Continuous You” exercise. |
| 3 | **Mindful living** | **Open acceptance**  **1. Overview：**  (1) Introduce the concept of acceptance and explain it using the metaphor “tug- of-war with monsters”;  (2) Outline the goals of acceptance and provide guidance on when to use it as a strategy.  **2. Practice：**  Guide nurses through “The Acceptance Tool Kit” exercises to learn how to accept anxiety and depression with a non-judgmental attitude, rather than trying to control or experientially avoid them.  **Contact with the present moment**  **1. Overview：**  (1) Introduce what it means to contact with the present moment, and use the metaphor “time machine” to explain it.  **2. Practice：**  Guide nurses through “mindfulness of your hand” exercise to enhance their awareness of the present moment and reduce feelings of regret about the past and fear about the future.  **Video duration: 27 minutes.** | **Struggling vs. opening up worksheet：** Nurses are encouraged to think about and answer the following questions.  (1) How long and to what extent do I struggle with unwanted emotions or feelings, and do I open to them?  (2) What is the long-term impact of the two different coping strategies of struggle and openness on my work life? | Mindfulness of your hand;  “The acceptance tool kit” exercises. |
| 4 | **Knowing what matters** | **Clarifying values**  **1.** **Overview：**  (1) Introduce the concept of values and explain it using the metaphor “compass”;  (2) Explain the difference between values and goal;  (3) Explain the four key points of values.  **2. Practice：**  Guide nurses through the “imagine your retirement party” exercise to help them clarify their core values and what is most important to them.  **Video duration: 15 minutes.** | **Values worksheet：**Nurses are encouraged to think about and answer the following questions.  (1) What is most important in my whole life?  (2) What kind of nurse do I want to be?  (3) What have I already done to achieve this? What do I plan to do next? | “Hexaflex” exercise;  “The acceptance tool kit” exercises. |
| 5 | **Doing important things** | **Committed action**  **1. Overview：**  (1) Introduce what committed action is, when to use it, and how to deal with obstacles (anxiety, depression, etc.);  (2) Summarize important factors to consider when using ACT, and encourage nurses to use ACT frequently to solve their own problems in both their work and life.  **2. Practice：**  Guide nurses to set values-based SMART (specific, meaningful, adaptive, realistic, time-framed) goals based on their own working conditions.  **Video duration: 15 minutes.** | **The willingness and action plan：** Nurses are encouraged to think about and answer the following questions.  (1) What are my current goals and goal-based values? What are my actions to achieve my goals? How to do it specifically?  (2) I’m willing to make room for some difficult, scary thoughts and feelings in taking action, not get caught up in them or fight them. I prefer to use ACT techniques to get out of trouble than to give up easily, so the time for me to take the first action step is? | “Hexaflex” exercise;  Mindful Breathing. |

Note. ACT=Acceptance and Commitment Therapy; The “Hexaflex” exercise lasted 19 minutes; The mindful breathing lasted 9 minutes; The “Continuous You” exercise lasted 10 minutes; The mindfulness of your hand lasted 10 minutes;“The acceptance tool kit” exercises lasted 10 minutes.
