# Supplementary figures and images for: Effects and Mechanisms of a Web- and Mobile-Based Acceptance and Commitment Therapy Intervention for Anxiety and Depression Symptoms in Nurses: Fully Decentralized Randomized Controlled Trial
Source: J Med Internet Res. 2023 Nov 27;25:e51549. doi: 10.2196/51549 (PMC10714267; doi:10.2196/51549)

**Multimedia Appendix 2**


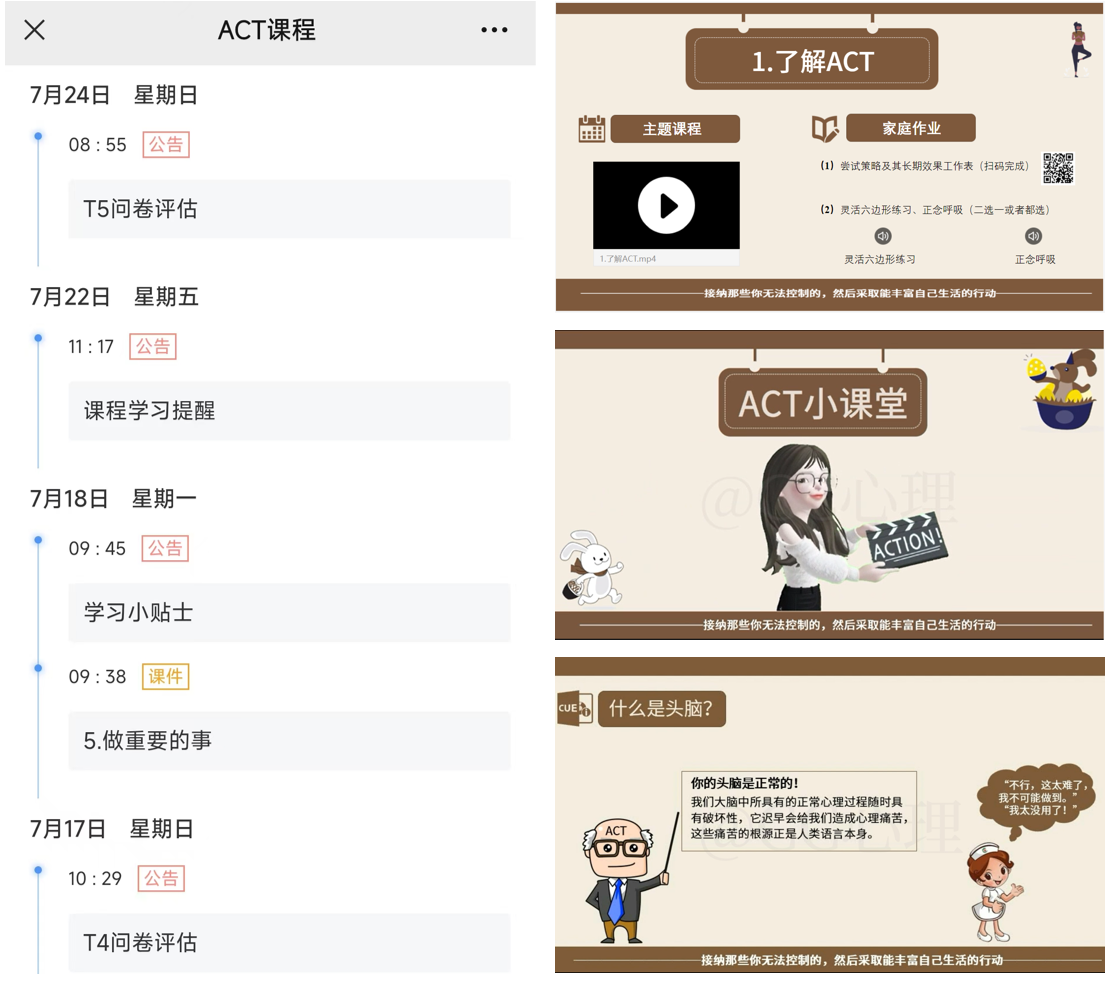


The screenshot of the Rain Classroom WeChat mini program

Supplement: Multimedia Appendix 2 [file jmir_v25i1e51549_app2.docx]
