# Supplementary material for: Effects and Mechanisms of a Web- and Mobile-Based Acceptance and Commitment Therapy Intervention for Anxiety and Depression Symptoms in Nurses: Fully Decentralized Randomized Controlled Trial
Source: J Med Internet Res. 2023 Nov 27;25:e51549. doi: 10.2196/51549 (PMC10714267; doi:10.2196/51549)
Supplement: Multimedia Appendix 3 [file jmir_v25i1e51549_app3.docx]

**Multimedia Appendix 3**

Sensitivity analysis was performed using the complete case data. The results showed that the depression symptom score at T3 was lower in the intervention group than in the control group, and the difference was statistically significant (estimated mean difference=-2.01, *P*=0.03). The work performance score at T5 was higher in the intervention group than in the control group, and the difference was statistically significant (estimated mean difference=2.95, *P*=0.02). Other results were consistent with those obtained through the intention-to-treat (ITT) analysis.

Effects of the web-based and mobile acceptance and commitment therapy intervention based on complete case data

| Outcome  and time | Estimated mean difference, mean (95% CI) | *P* | Group effect | | Time effect | | Group×time effect | |
| --- | --- | --- | --- | --- | --- | --- | --- | --- |
|  |  |  | Wald*χ^2^* | *P* | Wald*χ^2^* | *P* | Wald*χ^2^* | *P* |
| **Anxiety symptom** | | | | | | | | |
| T1 | -0.13(-1.54,1.27) | 0.85 | 4.39 | 0.04 | 51.29 | <0.001 | 14.56 | 0.01 |
| T2 | -0.57(-1.86,0.73) | 0.39 |  |  |  |  |  |  |
| T3 | -0.98(-2.42, 0.46) | 0.18 |  |  |  |  |  |  |
| T4 | -1.45(-2.92, 0.01) | 0.05 |  |  |  |  |  |  |
| T5 | -2.47(-1.09, -0.97) | <0.001 |  |  |  |  |  |  |
| T6 | -1.75(-3.32, -0.19) | 0.03 |  |  |  |  |  |  |
| **Depression symptom** | | | | | | | | |
| T1 | -0.25(-1.92,1.42) | 0.77 | 7.905 | 0.005 | 360.88 | <0.001 | 15.06 | 0.01 |
| T2 | -0.85(-2.46,0.75) | 0.30 |  |  |  |  |  |  |
| T3 | -2.01(-3.85, 0.17) | 0.03 |  |  |  |  |  |  |
| T4 | -1.66(-2.84, -0.49) | 0.005 |  |  |  |  |  |  |
| T5 | -3.07(-4.82, -1.33) | 0.001 |  |  |  |  |  |  |
| T6 | -3.31(-5.19, -1.42) | 0.001 |  |  |  |  |  |  |
| **Sleep quality** | | | | | | | | |
| T1 | -0.06(-0.88,1.01) | 0.90 | 12.15 | <0.001 | 36.04 | <0.001 | 28.03 | <0.001 |
| T5 | -2.11(-3.10, -1.13) | <0.001 |  |  |  |  |  |  |
| T6 | -2.42(-3.50, -1.33) | <0.001 |  |  |  |  |  |  |
| **Job burnout** | | | | | | | | |
| T1 | -1.69(-9.04,5.65) | 0.65 | 5.53 | 0.02 | 18.42 | <0.001 | 9.00 | 0.011 |
| T5 | -8.85(-16.08, -1.63) | 0.02 |  |  |  |  |  |  |
| T6 | -12.02(19.01, -5.02) | 0.001 |  |  |  |  |  |  |
| **Work performance** | | | | | | | | |
| T1 | 0.26(-1.95,2.47) | 0.82 | 5.22 | 0.02 | 2.85 | 0.24 | 11.88 | 0.003 |
| T5 | 2.95(0.44,5.46) | 0.02 |  |  |  |  |  |  |
| T6 | 3.92(1.23, 6.61) | 0.004 |  |  |  |  |  |  |
