# Supplementary material for: Effects and Mechanisms of a Web- and Mobile-Based Acceptance and Commitment Therapy Intervention for Anxiety and Depression Symptoms in Nurses: Fully Decentralized Randomized Controlled Trial
Source: J Med Internet Res. 2023 Nov 27;25:e51549. doi: 10.2196/51549 (PMC10714267; doi:10.2196/51549)
Supplement: Multimedia Appendix 4 [file jmir_v25i1e51549_app4.docx]

**Multimedia Appendix 4**

Comparison of baseline general information for completed and missed samples

| Variable | Missing sample (n=36) | Completed sample (n=109) | *χ*^2^ | *P* |
| --- | --- | --- | --- | --- |
|  | n (%) | n (%) |  |  |
| **Age** |  |  | 0.683 | 0.41 |
| <35 | 14(38.9) | 51(46.8) |  |  |
| ≥35 | 22(61.1) | 58(53.2) |  |  |
| **Sex** |  |  | 0.000 | 0.99 |
| Male | 1(2.8) | 3(2.8) |  |  |
| Female | 35(97.2) | 106(97.2) |  |  |
| **BMI** |  |  | 0.448 | 0.50 |
| Normal | 25(69.4) | 69(63.3) |  |  |
| Abnormal | 11(30.6) | 40(36.7) |  |  |
| **Average monthly income** |  |  | 0.278 | 0.60 |
| <8000 yuan | 17(47.2) | 46(42.2) |  |  |
| ≥8000 yuan | 19(52.8) | 63(57.8) |  |  |
| **Marital status** |  |  | 0.020 | 0.89 |
| Single | 8(22.2) | 23(21.1) |  |  |
| Married | 28(77.8) | 86(78.9) |  |  |
| **Education** |  |  | 0.461 | 0.50 |
| Bachelor’s or lower degree | 29(80.6) | 93(85.3) |  |  |
| Master’s or higher degree | 7(19.4) | 16(14.7) |  |  |
| **Working department** |  |  | 0.483 | 0.92 |
| Internal medicine | 13(36.1) | 35(32.1) |  |  |
| Surgery | 6(16.7) | 18(16.5) |  |  |
| Gynecology and pediatrics | 4(11.0) | 10(9.2) |  |  |
| Other | 13(36.1) | 46(42.2) |  |  |
| **Employment type** |  |  | 0.859 | 0.65 |
| Authorized strength | 20(55.6) | 51(46.8) |  |  |
| Human agency | 3(8.3) | 12(11.0) |  |  |
| Other | 13(36.1) | 46(42.2) |  |  |
| **Type of nursing role** |  |  | 0.302 | 0.58 |
| Nurse practitioner or below | 13(36.1) | 45(41.3) |  |  |
| Supervisor or above | 23(63.9) | 64(58.7) |  |  |
| **Position** |  |  | 0.001 | 0.98 |
| Nurse | 27(75.0) | 82(75.2) |  |  |
| Head nurse or above | 9(25.0) | 27(24.8) |  |  |
| **Working years** |  |  | 0.848 | 0.36 |
| ≤10 years | 14(38.9) | 52(47.7) |  |  |
| ＞10 years | 22(61.1) | 57(52.3) |  |  |
| **Weekly working hours** |  |  | 0.174 | 0.68 |
| ≤40h/week | 8(22.2) | 28(25.7) |  |  |
| >40h/week | 28(77.8) | 81(74.3) |  |  |
| **Number of night shifts per month** |  |  | 1.979 | 0.37 |
| 0 | 19(52.8) | 45(41.3) |  |  |
| 1-4 | 7(19.4) | 20(18.3) |  |  |
| ≥5 | 10(27.8) | 44(40.4) |  |  |

Note. BMI=body mass index.
